# Lung and heart-lung transplantation in pulmonary arterial hypertension

**DOI:** 10.1371/journal.pone.0187811

**Published:** 2017-11-21

**Authors:** Manuel López-Meseguer, Carlos A. Quezada, Maria A. Ramon, María Lázaro, Laura Dos, Antonio Lara, Raquel López, Isabel Blanco, Pilar Escribano, Antonio Roman, on behalf of the REHAP Investigators

**Affiliations:** 1 Department of Pneumology, Hospital Universitari Vall d'Hebron, Barcelona, Spain; 2 Departament de Medicina, Universitat Autònoma de Barcelona (UAB), Barcelona, Spain; 3 Biomedical Research Networking Center on Respiratory Diseases (CIBERES), Madrid, Spain; 4 Pulmonary Hypertension Unit. Cardiology Department, Hospital Universitario doce de Octubre. Madrid, Spain; 5 Department of Cardiology. Hospital Virgen de la Salud, Toledo, Spain; 6 Department of Cardiology. Unidad de C.C. Adolescente y Adulto Vall d'Hebron-Sant Pau. Barcelona, Spain; 7 Department of Cardiology. Hospital Universitario de Canarias. Santa Cruz de Tenerife, Spain; 8 Department of Pneumonology. Hospital Universitario La Fe, Valencia, Spain; 9 Department of Pulmonary Medicine, Hospital Clínic-IDIBAPS, University of Barcelona. Barcelona, Spain; 10 University of Barcelona. Barcelona, Spain; Stanford University, UNITED STATES

## Abstract

**Background:**

Real use of lung (LT) and heart-lung (HLT) transplantation in pulmonary arterial hypertension (PAH) is unknown. The objectives were to describe the indication of these procedures on PAH treatment in a national cohort of PAH patients, and to analyze the potential improvement of its indication in severe patients.

**Methods:**

Eligibility for LT/HLT was assessed for each deceased patient. Incident patients from REHAP diagnosed between January 2007 and March 2015 and considered eligible for LT/HLT were grouped as follows: those who finally underwent transplantation (LTP) and those who died (D-Non-LT).

**Findings:**

Of 1391 patients included in REHAP, 36 (3%) were LTP and 375 (27%) died. Among those who died, 36 (3%) were D-Non-LT. LTP and D-Non-LT were equal in terms of age, gender, and clinical status. Ten percent of those who died were functional class I-II. Patients functional class IV were less likely to undergo LT (8.3% LTP vs. 30.6% D-Non-LT, p = 0.017). Patients with idiopathic and drug/toxin-associated PAH were more likely to undergo LT (44.4% LTP vs. 16.7% D-Non-LT, p = 0.011).

**Conclusions:**

The present results show that the use of LT/HLT could double for this indication. Relevant mortality in early functional class reflects the difficulties in establishing the risk of death in PAH.

## Introduction

Pulmonary arterial hypertension (PAH) is a severe and life-threatening condition with high mortality [1–3]. Double lung and heart-lung transplantation must be considered in all patients with severe PAH who have an inadequate response to optimal specific drug therapy [4,5], even though LT is used infrequently in PAH, with only about 100 procedures performed yearly worldwide [6]. In patients with PAH, limited availability of organs leads to higher waiting list mortality. It has been quantified at 19% per year since the implementation of the *lung allocation score*, a mortality that is significantly higher than for the rest of the diseases with indication for lung transplantation (LT)/heart lung transplantation (HLT) [7,8] for which early referral strategies were developed in order to minimize the effect of the limitations imposed by organ availability [6,9]. Although bridging with extracorporeal support systems can potentially minimize mortality on the waiting list, elective LT continues to be the optimal strategy and, when possible, must be the first choice for transplant candidates [6,10]. We hypothesized that the impact of LT on the global mortality of PAH is limited.

The objectives of this study were to evaluate the impact of LT/HLT on the treatment of PAH in a national cohort of incident PAH patients recruited over a long period and to analyse the potential increase in the use of this procedure in severe patients.

## Methods

### REHAP

The characteristics of the Spanish Registry of Pulmonary Arterial Hypertension (REHAP), which provides the most valuable epidemiological data for PAH in Spain, have been described elsewhere [3]. Briefly, REHAP is a voluntary registry comprising 52 Spanish hospitals that is designed to prospectively collect exhaustive information on the demographics, management, and outcome of patients newly and previously diagnosed with PAH and chronic thromboembolic pulmonary arterial hypertension (CTEPH). REHAP was launched in January 2007, and all patients diagnosed from that date were prospectively recruited and classed as incident patients.

### Design and population

In the present study, we retrospectively analysed all incident PAH patients who died or underwent LT between January 2007 and March 2015. Non-operable CTEPH patients were included in the analysis. Patients with human immunodeficiency virus (HIV) infection and portopulmonary hypertension were excluded, as they were not candidates for LT. Data from REHAP were contrasted with data from the Spanish National Transplant Organization.

**Fig 1:**
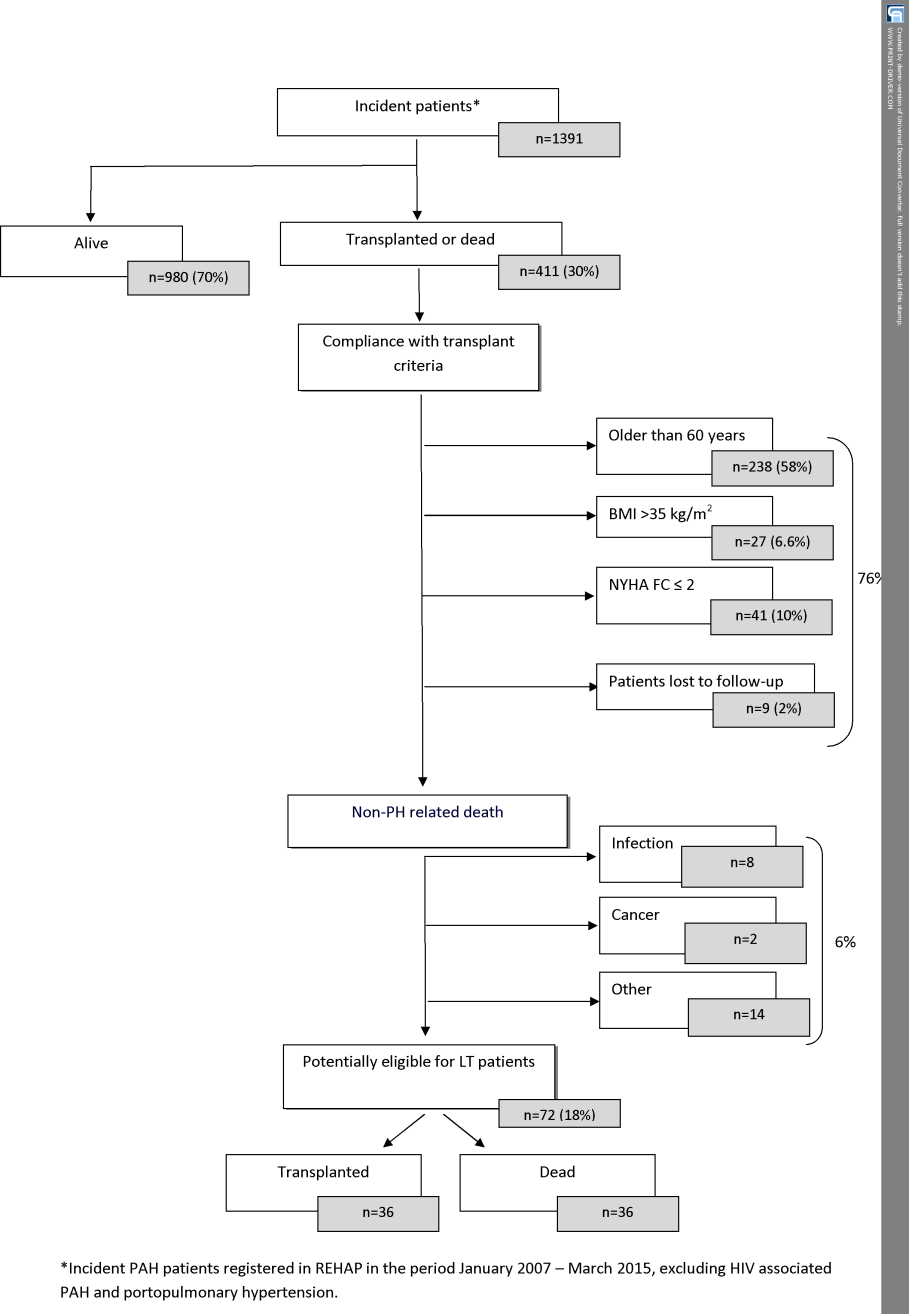
Flowchart of a national cohort of 1391 incident PAH and CTEPH patients, focusing on those who died or underwent LT and considering their eligibility for LT between January 2007 and March 2015.

Death and LT were considered the final outcomes of PAH. Eligibility for LT was assessed in patients who died and patients who underwent LT. For the purposes of this study, the results obtained after a maximum of 3–4 months before the event were used to establish the patient’s clinical situation near LT or death. Almost all of the absolute and relative contraindications for LT are shown in Fig 1. Patients with obvious contraindications for LT and those who died from non–PAH-related causes were considered not eligible for LT. The remaining 72 patients were considered potentially eligible for LT and were grouped as follows: patients who underwent LT (LTP); patients who died but were potentially eligible for LT (D-Non-LT). D-Non-LT were considered eligible for LT if they were New York Heart Association (NYHA) functional class III or IV, as recorded in the REHAP 3 to 4 months before death, and died from a PAH-related cause. Patients who died from a cause not related to PAH, those NYHA class I or II, and obese patients (BMI >35 kg/m^2^) were considered not eligible for LT [10]. Patients with pulmonary veno-occlusive disease (PVOD) were considered eligible for LT regardless of their functional class. Information about other comorbidities that could have influenced eligibility was not available.

All patients signed the informed consent at the moment of inclusion in the REHAP Registry giving permission for research purposes. The National Registry successfully passed quality controls and specific Ethic Committee and IRB approval.

### Statistical analysis

Qualitative data are expressed as frequencies and percentages. Normally distributed quantitative data are expressed as mean±standard deviation (SD); non-normally distributed data are expressed as median and interquartile range (IQR). The demographic and clinical characteristics of LTP and D-Non-LT were compared using an unpaired *t* test, Mann Whitney test, chi-square test, or Fisher exact test, as appropriate. Time from diagnosis to transplantation or death was assessed in both groups using Kaplan-Meier curves and the log-rank test. The analysis was conducted using Stata 12.1 (StataCorp, College Station, Texas, USA). A p value <0.05 was considered significant.

## Results

Between January 2007 and March 2015, a total of 1391 incident PAH patients (excluding portopulmonary and HIV-related PAH patients) and non-operable CTEPH patients were included in REHAP; 375 (27%) reached the final outcome of death and 36 (3%) received a LT. Among those who died, 306 (82%) died of a non–PAH-related cause or did not meet the established criteria for LT. In this subgroup, 58% of the patients were considered too old, 7% were obese, and 10% were NYHA class I-II (Fig 1). Twenty-four patients among those who died (6%), died from a non–PAH-related cause and were not considered eligible for LT (infection, 8; cancer, 2; other, 14). In other 9 (2%) of the dead patients, the eligibility for LT could not be assessed because of the significant lack of data. Finally, of 72 patients who were potentially eligible for LT, 36 died and 36 were transplanted.

Table 1 presents the main clinical and functional characteristics of these 72 patients. Comparison of transplanted and non-transplanted patients showed that both groups were equal in terms of age, gender, and clinical status measured in all its dimensions. Pulmonary function was analysed by: forced vital capacity (FVC), forced expiratory volume in the first second (FEV_1_), total lung capacity (TLC), pulmonary diffusion capacity (DLCO), peripheral capillary oxygen saturation (SpO_2_), partial pressure of oxygen (pO_2_), partial pressure of carbon dioxide (pCO_2_). Exercise capacity was represented by the 6-minute walking test (6mWT) and echocardiographic measure of pulmonary systolic pressure (sPAP) and tricuspid annular plane systolic excursion (TAPSE) were also analysed. Both groups were also compared by their hemodynamic status measured by mean pulmonary artery pressure (mPAP), mean capillary wedge pressure (mCWP), right atrial pressure (RAP), pulmonary vascular resistance (PVR), cardiac output (CO) and cardiac index (CI).

**Table 1:** Characteristics of PAH patients who were potentially eligible for LT stratified by transplantation or death during follow-up (n = 72).

	All* n = 72	Transplantation n = 36	Death n = 36	p
**Sociodemographic variables**				
Age (years), mean (SD)	41.0 (14.4)	39.6 (14.1)	42.6 (14.7)	0.413
Age ≥50 years, n (%)	25 (34.7)	9 (25.0)	16 (44.4)	0.083
Gender: Women, n (%)	49 (68.1)	24 (66.7)	25 (66.4)	0.800
**Clinical variables**				
Etiology, n (%)				
Idiopathic, heritable and drug-associated	22 (30.6)	16 (44.4)	6 (16.7)	0.011
Connective tissue disease	17 (23.6)	6 (16.7)	11 (30.6)	0.165
PVOD	8 (11.1)	6 (16.7)	2 (5.6)	0.134
CTEPH	5 (6.9)	1 (2.8)	4 (11.1)	0.375
Congenital shunts	16 (22.2)	4 (11.1)	12 (33.3)	0.045
Other	4 (5.6)	3 (8.3)	1 (2.8)	0.614
NYHA functional class 4, n (%)	14 (19.4)	3 (8.3)	11 (30.6)	0.017
**Pulmonary function tests**				
FVC (%pred), mean (SD)	78.3 (20.7)	82.3 (19.7)	74.0 (21.2)	0.114
FEV_1_ (%pred), mean (SD)	76.1 (18.4)	77.9 (19.3)	74.2 (18.4)	0.443
TLC (%pred), mean (SD)	85.0 (17.9)	86.1 (20.8)	83.7 (19.7)	0.282
DLCO (%pred), mean (SD)	46.4 (20.3)	44.6 (19.1)	49.5 (22.9)	0.465
SpO_2_ (%), mean (SD)	90.8 (5.9)	92.1 (4.7)	89.4 (6.5)	0.192
PO_2_ (mmHg), mean (SD)	61.1 (14.1)	64.2 (13.8)	57.8 (14.1)	0.119
PCO_2_ (mmHg), mean (SD)	32.8 (5.6)	32.0 (5.4)	33.8 (5.8)	0.275
6MWT (metres), median (IQR)	345 (243–439)	363 (240–432)	320 (247–445)	0.711
**Echocardiography**				
sPAP (mmHg), mean (SD)	85.9 (20.5)	84.1 (21.3)	87.6 (20.5)	0.501
TAPSE (mm), mean (SD)	16.1 (5.0)	16.3 (4.9)	15.8 (5.2)	0.748
Pericardial effusion n (%)	21 (30.4)	8 (22.9)	13 (38.2)	0.197
**Cardiac catheterization (baseline)**				
CO (L/min), mean (SD)	3.9 (1.3)	4.1 (1.3)	3.5 (1.4)	0.114
CI (L/min/m^2^), mean (SD)	2.3 (0.7)	2.3 (0.7)	2.3 (0.7)	0.757
RAP (mmHg), mean (SD)	11.9 (6.7)	12.0 (7.0)	11.7 (6.4)	0.968
mPAP (mmHg), mean (SD)	57.2 (15.2)	58.7 (16.8)	55.4 (12.9)	0.3926
mCWP (mmHg), mean (SD)	10.2 (3.5)	10.3 (3.7)	10.1 (3.1)	0.817
PVR (Wood units), median (IQR)	11.9 (8.0–19.8)	11.1 (8.0–18.6)	12.7 (8.0–20.2)	0.398
**Follow-up**				
Time from diagnosis to death or LT (days), median (IQR)	473 (258–1178)	473 (204–780)	450 (264–1294)	0.588
Follow-up < 1 month, n (%)	5 (6.9)	1 (3.0)	4 (10.5)	0.358
Follow-up < 3 month, n (%)	9 (12.5)	5 (13.9)	4 (11.1)	0.722
Follow-up < 1 year, n (%)	26 (36.1)	13 (36.1)	13 (36.1)	0.999

SD, standard deviation; PVOD, pulmonary veno-occlusive disease; CTEPH, chronic thromboembolic pulmonary hypertension; NYHA, New York Heart Association; FVC, forced vital capacity; FEV_1_, forced expiratory volume in the first second; TLC, total lung capacity; DLCO, pulmonary diffusion capacity; SpO_2_, peripheral capillary oxygen saturation; pO_2_, partial pressure of oxygen; pCO_2_, partial pressure of carbon dioxide; 6MWT, 6-minute walking test; sPAP, systolic pulmonary artery pressure; TAPSE, tricuspid annular plane systolic excursion; CO, cardiac output; CI, cardiac index; RAP, right atrial pressure; mPAP, mean pulmonary artery pressure; mCWP, mean capillary wedge pressure; PVR, pulmonary vascular resistance; IQR, interquartile range. WU: wood units.

*Values were missing in some variables as follows: 10 in FVC, 10 in FEV_1_, 25 in TLC, 30 in DLCO, 24 en SpO_2_, 25 in PO_2_, 25 in PCO_2_, 12 in 6MWD, 7 in PAP, 21 in TAPSE, 3 in pericardial effusion, 10 in cardiac output, 22 in cardiac index, 7 right atrial pressure, 9 in dPAP, 8 in mPAP, 7 in capillary wedge pressure, 10 in pulmonary vascular resistance.

With respect to aetiology, patients with idiopathic, heritable, and drug/toxin-associated PAH were more likely to undergo LT (44% LTP vs. 17% D-Non-LT, p = 0.011), while patients with PAH associated with congenital heart disease were less likely to undergo LT (11.1% LTP vs. 33.3% D-Non-LT, p = 0.045). Of the 5 (7%) CTEPH patients considered eligible for LT, 1 eventually received a transplant. Patients NYHA class IV were less likely to undergo LT than patients NYHA class III (8% LTP vs. 31% D-Non-LT, p = 0.017).

**Fig 2:**
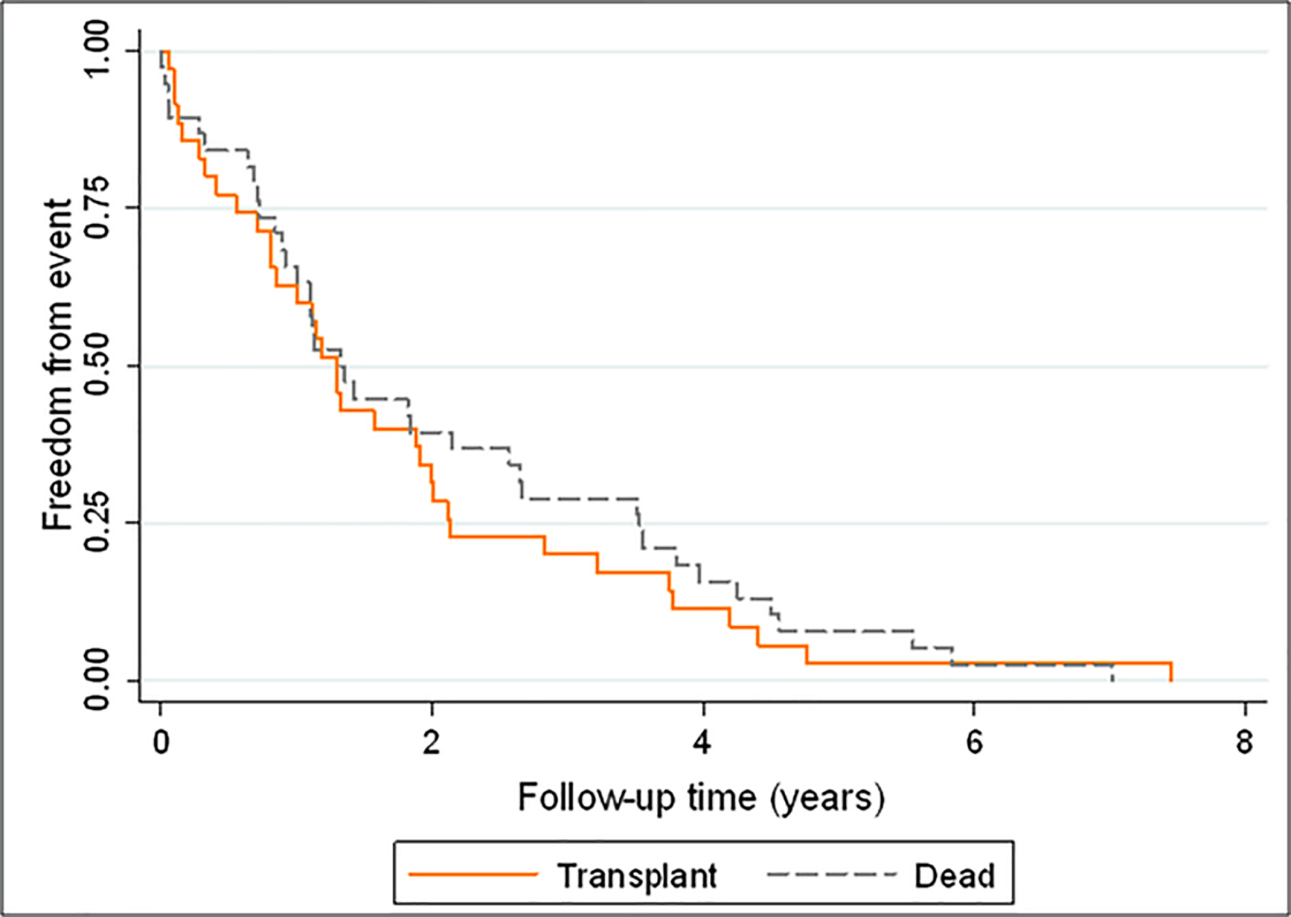
Mantel-Haenszel (log-rank) test. Time from diagnosis (diagnostic catheterization) to transplant or death did not differ during follow-up (p = 0.514).

Fig 2 shows the median follow-up from diagnosis to death or LT (473 days in LTP and 450 days in D-Non-LT, p = 0.514).

**Fig 3:**
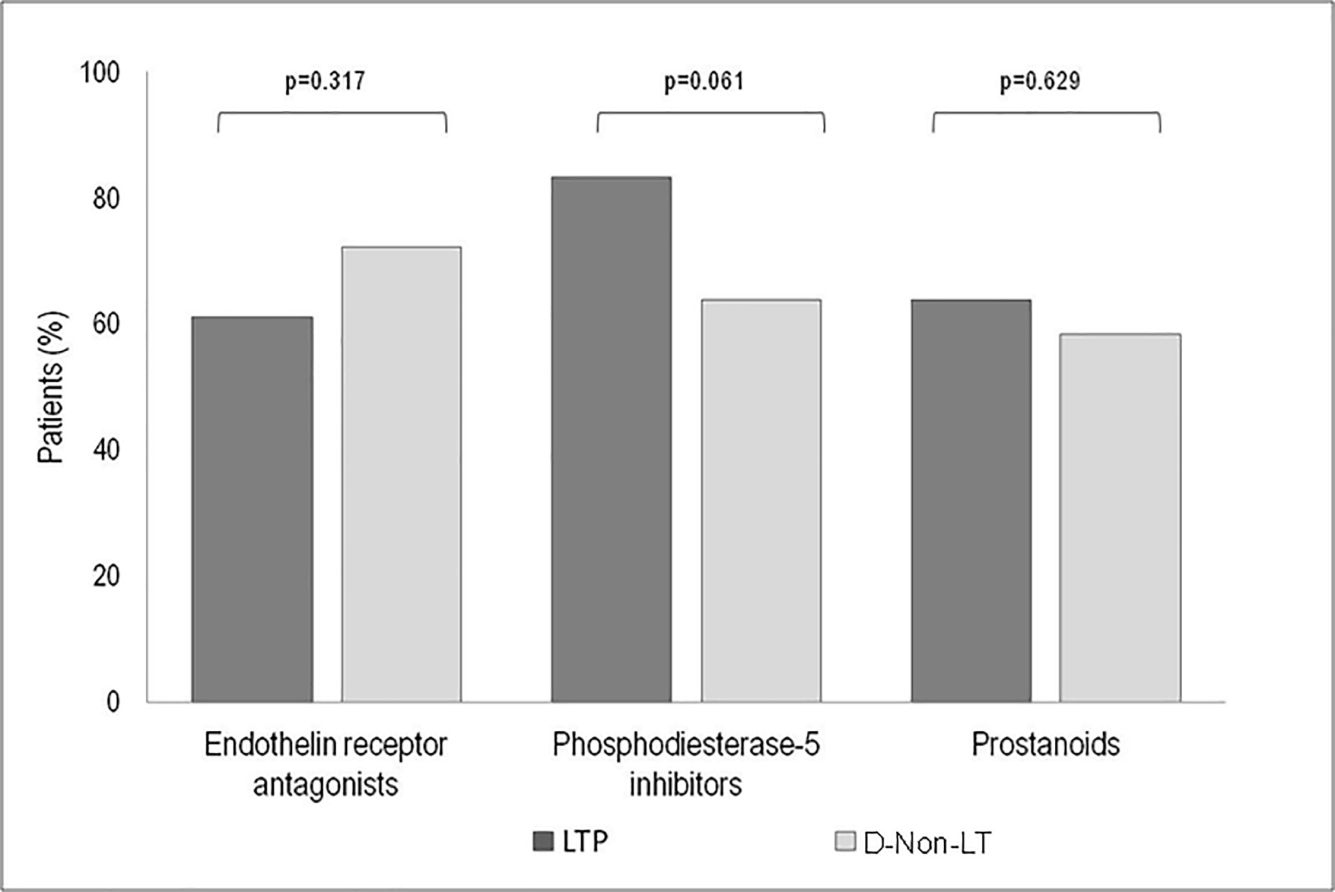
Use of PAH-targeted therapy in incident PAH and non-operable CTEPH patients who died or underwent LT between January 2007 and March 2015. Comparison between groups.

Pharmacological treatment is summarized in Fig 3. In the month before the event, 42 (58%) patients were receiving ≥2 specific PAH drugs or parenteral prostanoids, 24 (33%) were receiving monotherapy (1 inhaled iloprost, 1 sitaxentan, 1 tadalafil, 8 bosentan, and 13 sildenafil), and 6 (8%) were not receiving a specific PAH drug. Sildenafil was more frequently prescribed in LTP (81% vs. 53%, p = 0.012). There were no differences between groups in terms of prostanoids (65% for LTP and 61% for D-Non-LT), combined treatment, or parenteral prostanoids. As for treatment of pulmonary veno-occlusive disease, 2 out of 8 patients were receiving prostanoids (1 in monotherapy and 1 in combination with bosentan), 4 sildenafil in monotherapy, 1 bosentan plus sildenafil, and 1 no PAH-specific drugs.

**Fig 4:**
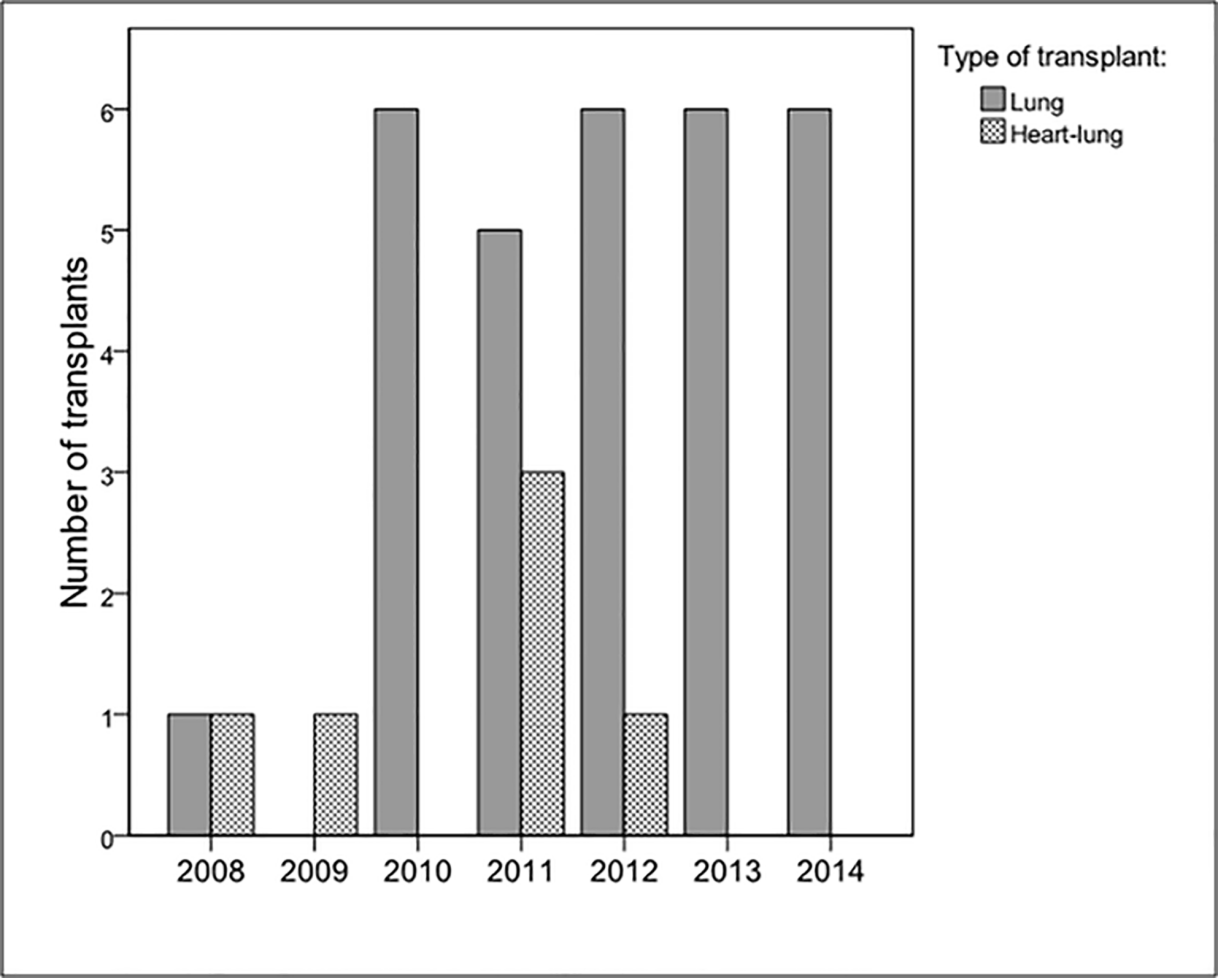
Yearly transplant activity by type of procedure (n = 36).

The most common technique was bilateral LT (83%), followed by combined heart and lung transplant (17%). No single LTs were recorded (Fig 4).

Considering the whole group of 72 potentially eligible patients, 47 (65%) patients were originally from LT centres (28 LTP [78%] and 19 D-Non-LT [53%]), thus showing that patients from LT centres were more likely to undergo transplantation than other patients.

## Discussion

The present study provides reliable clinical data on all deaths in a national cohort of incident PAH patients followed prospectively for a long period of time, thus enabling an evaluation of LT activity. Only 2.6% of the patients underwent LT/HLT. A limited impact of LT on mortality in the incident population of REHAP is observed, namely, only 36 (9%) of the 411 patients included in the study underwent LT. This result might reflect underuse of LT, which is a well-established therapy for severe PAH [4,5]. Retrospective analysis of eligibility in all dead patients was based on the fact that almost all relevant contraindications for LT are collected in REHAP. Exceeding the age limit was the main reason for exclusion from LT, with 58% of patients aged over 60 years. This result is consistent with current knowledge, since PAH has been redefined as a disease of the elderly in recent years [11], in contrast with reports from the early 1990s, when PAH was diagnosed mainly in younger individuals [12] Consequently, most PAH patients are not eligible for LT as they exceed the age limit. Although less strict criteria for inclusion on the LT waiting list were recently implemented and double LT is currently performed in patients aged ≥ 65 years, age limit will remain an unsolvable limitation to LT in PAH patients. Other relevant contraindication for LT in our population was obesity (7%), although its incidence is in the lower range compared with other countries [13]. Ten percent of the deaths occur in early NYHA functional class, which underlines the complexity of prognostic assessment in PAH. Furthermore, 6% of patients died from a non–PAH-related cause. Finally, only half of the potentially eligible patients underwent LT/HLT and, in the best scenario, if all eligible patients would have undergone LT, this would mean a 5.2% of the entire incident PAH population. These results are consistent with the fact that real use of LT for most respiratory diseases is limited and only a small number of patients receive a LT as a treatment [6]. The epidemiologic assessment of this issue is challenging, because reports on mortality and comorbidity in most respiratory diseases are scarce and most approaches are imprecise. The fact that only a few thousand of all patients with severe respiratory diseases undergo LT every year in developed countries enables us to conclude that the impact of LT on the treatment of severe chronic respiratory failure is very limited [10]. Our results are consistent with this observation and provide reliable epidemiological data; in REHAP, only 36 of 1391 incident patients (2.6%) underwent LT.

A relevant issue is how to increase eligibility for LT in PAH. Our study concludes that half of all potential candidates for LT did not get it, although this value may be underestimated, because non-reported comorbidities could have conditioned eligibility in some cases. Even so, the most optimistic approach shows that at least 18% of the incident patients who died would have been eligible for LT. Infrequent use of LT was reported in the late 1990s in Europe [14] and the United States [15] in patients who were receiving intravenous epoprostenol and among whom only 15% and 6.8%, respectively, underwent LT. In the analysis of survival in PAH in the modern era based on data from the French national registry, Humbert et al. also reported infrequent use of LT [2]. Over a period of 3 years, only 1 out of 56 (1.8%) consecutive incident patients with idiopathic, heritable, and anorexigen-associated PAH underwent LT, regardless of the functional class, whereas 25 patients died during the same period. This low frequency of LT in a group of incident patients who exhibited relevant mortality was explained by a shortage of organ donors. If we accept that a donor shortage impacts waiting list mortality for PAH (11,5% in Spanish PAH patients during the study period [data from the Spanish National Transplant Organization]), other possible reasons should be sought for the underuse of LT in patients with PAH in our setting, where donation and LT activity rates are high and waiting list mortality low [16].

Current guidelines recommend LT/HLT for severe PAH. LT has a precise and well-defined place in the PAH treatment algorithm, which stresses the importance of eligibility for LT [5]. Patients should be considered eligible for LT if the response to first-line therapy is not as expected and should be included on the waiting list if their clinical status remains unsatisfactory, that is, persistent NYHA class III-IV despite receiving optimal medical therapy containing parenteral prostanoids [5,14,15]. The observed underuse of prostanoids in LTP and D-Non-LT patients is in accordance with data from other national registries[17]. This underlines the idea that prostacyclin treatment could be considered too late for many clinicians as they need a complex infrastructure to proceed with this therapy, so this is a clear area of improvement in the clinical management of PAH. This strategy aims to minimize waiting list mortality and to enable an elective LT strategy in most cases, thus guaranteeing the best possible outcomes. The fact that patients NYHA class IV are less likely to undergo LT would reflect a tendency towards excluding very symptomatic patients from LT, which goes against the guidelines [5]. This tendency may be explained in part by the considerable difficulties involved in bridging patients with severe PAH to LT. In the 1990s, parenteral prostanoids were considered an acceptable bridge for LT [18,19], whereas in recent years, bridging strategies for LT have evolved towards extracorporeal respiratory and circulatory support systems [20, 21]. Underuse of LT in patients NYHA class IV could be explained by the fact that this technology, which requires a very well-trained and dedicated team, is only beginning to be used in Spain. It is uncertain whether these new techniques will change this scenario in the near future.

The relevant percentage of patients who died during the first 3 months after diagnosis (22%) reflects a phenotype of rapid deterioration that complicates the decision to treat with LT. This *non-responder to any treatment* phenotype was first described in the early years of the last decade [14,15] and makes inclusion on the LT waiting list extremely challenging. In the present study, time since diagnosis was the same for LTP and D-Non-LT. The shortest time from diagnosis of PAH to LT was less than 1 month (in a patient with pulmonary veno-occlusive disease), and 17% of LTP received their transplant within 3 months of their diagnosis. Moreover, during the study period, 27% of PAH patients in Spain were included on an urgent LT waiting list with a short mean waiting time of 14 (1–71) days (data from the Spanish National Transplant Organization). Consequently, decisions in this group can be taken quickly. We must presume that the eventual lack of eligibility in half of the potentially eligible patients could have been motivated by comorbidities not collected in REHAP or that LT could have been considered late or not at all. The latter possibility is supported by differences in the indication for LT between centres. LT centres tended to advocate transplant more than non-LT centres: 60% of eligible patients in LT centres underwent LT, compared with only 32% in non-LT centres, although the percentages reported could be subject to selection bias. Therefore, this finding reinforces the recommendation that potentially eligible PAH patients should be referred early to LT centres [5].

Our greatest concern was how to identify patients who are near death and should be potentially eligible for LT. We found that 41 (10%) patients died unexpectedly, as their NYHA functional class was considered low. Although their eligibility cannot be ruled out—some could have been misclassified, had poor prognostic factors not available in REHAP, or died of a non–PAH-related cause—PAH is clearly a well-established cause of unexpected death, even in the early stages [22]. This problem remains unsolved, despite the development of scores to better assess the risk of death in patients with PAH [23,24]. By contrast, recent data on long-term mortality suggest that functional class is the strongest prognostic factor. Five-year mortality in the REVEAL Registry ranged from 20% to 29% for patients NYHA class II at diagnosis [25]. We must accept that there is a lack of reliable tools that enable us to identify patients who die with a low NYHA functional class. There is a clearly unmet need for better prognostic factors that enable us to act early and optimize the treatment of these patients.

Although a significant proportion of patients were receiving combined therapy or parenteral prostanoids, they all remained NYHA class III or IV, thus confirming their severity and justifying the indication for LT. These results could reflect a non-aggressive approach in many centres for patients who are about to die. Similar results have been reported by other national registries despite the recommendations of current guidelines [13].

The strengths of this study are its large sample of PAH patients, which is considered to be representative of the epidemiology of PAH in Spain [3], full characterization of incident patients, long follow-up, and the clinical relevance of the issue addressed. However, our findings are also subject to limitations. First, since REHAP is a voluntary registry, not all patients diagnosed in Spain during the study period were included. Nevertheless, REHAP estimates an overall prevalence of PAH in Spain of 19.2 cases per million adult inhabitants, which is very similar to that reported by other national registries [3,26,27]. Second, despite the accurate and extensive data available, we did not perform a more detailed search for the reasons why the 36 D-Non-LT patients were not included or consider the possibility that some patients could have rejected LT.

In conclusion, a low proportion of incident and severe PAH patients underwent LT during the first eight years of existence of the Spanish National Registry. This finding cannot only be explained by the scarcity of donors and the inherent epidemiological characteristics of PAH must also be taken into account. Establishing the risk of death and the need for LT in stable patients with a low functional class is an unresolved problem. Therefore, access to LT in PAH should be considered at early stages to maximize the possibility of transplant for potential candidates.
