# Correction: Lung and heart-lung transplantation in pulmonary arterial hypertension

**DOI:** 10.1371/journal.pone.0192100

**Published:** 2018-01-29

**Authors:** Manuel López-Meseguer, Carlos A. Quezada, Maria A. Ramon, María Lázaro, Laura Dos, Antonio Lara, Raquel López, Isabel Blanco, Pilar Escribano, Antonio Roman, on behalf of the REHAP Investigators

The member list of the REHAP Investigators is missing from the Acknowledgements section. The full member list has been included in the Supporting Information file S1 File.

## Supporting information

S1 File REHAP registry members. (DOC)
