# Supplementary material for: Correction: Lung and heart-lung transplantation in pulmonary arterial hypertension
Source: PLoS One. 2018 Jan 29;13(1):e0192100. doi: 10.1371/journal.pone.0192100 (PMC5788393; doi:10.1371/journal.pone.0192100)
Supplement: S1 File — (DOC) [file pone.0192100.s001.doc]

**REHAP REGISTRY MEMBERS**

M. Alcasena 1; L. Almenar 2; P. Álvarez 3; J. Barbera 4; E. Barrios 5; P. Bedate 6; A. Campo 7; I. Casado 8; A. Castro 9; J. Cifrián 10; J. Domingo 11; L. Dos 12; V. Egurbide 13; T. Elías 14; P. Escribano 15; A. Fortuna 16; I. Furest 17; P. Gallego 18; S. García 19; F. García 20; E. Garrido-Lestache 21; J. Gaudo 22; J. Gil 23; F. Guerra 24; J. Jiménez 25; G. Juan 26; A. Lara 27; M. Lázaro 28; R. López 29; M. López 30; M. Marín 31; E. Martínez 32; F. Martínez 33; F. Mazo 34; L. Molina 35; T. Mombiela 36; I. Otero 37; F. Pastor 38; G. Pérez 39; P. Ramírez 40; A. Román 41; J. Rueda 42; E. Sala 43; J. Segovia 44; V. Suberviola 45;

1. Servicio de Cardiología. Hospital de Navarra - Pamplona (Navarra)

2. Servicio de Cardiología. Hospital Universitario y Politécnico La Fe - Valencia (Valencia)

3. Servicio de Neumología. Complejo Asistencial Universitario de Salamanca (CAUSA) - Salamanca (Salamanca)

4. Servicio de Neumología. Hospital Universitario Clínic de Barcelona - Barcelona (Barcelona)

5. Servicio de Cardiología. Hospital Universitario Rey Juan Carlos - Móstoles (Madrid)

6. Servicio de Neumología. Hospital Universitario Central de Asturias - Oviedo (Asturias)

7. Servicio de Neumología. Clínica Universidad de Navarra (Sede Pamplona) - Pamplona (Navarra)

8. Servicio de Neumología. Hospital Universitario Virgen de las Nieves - Granada (Granada)

9. Servicio de Cardiología. Hospital Universitario Virgen Macarena - Sevilla (Sevilla)

10. Servicio de Neumología. Hospital Universitario Marqués de Valdecilla - Santander (Cantabria)

11. Servicio de Neumología. Hospital Universitario Miguel Servet - Zaragoza (Zaragoza)

12. Servicio de Cardiología. Hospitales Universitarios Vall d'Hebron - Sant Pau - Barcelona (Barcelona)

13. Servicio de Medicina Interna. Hospital Universitario Cruces - Barakaldo (Vizcaya)

14. Servicio de Neumología. Hospital Universitario Virgen del Rocío - Sevilla (Sevilla)

15. Servicio de Cardiología. Hospital Universitario 12 de Octubre - Madrid (Madrid)

16. Servicio de Neumología. Hospital Universitario de la Santa Creu i Sant Pau - Barcelona (Barcelona)

17. Servicio de Neumología. Hospital Universitario Dr. Peset - Valencia (Valencia)

18. Servicio de Cardiología. Hospital Universitario Virgen Macarena - Sevilla (Sevilla)

19. Servicio de Neumología. Hospital de León - León (León)

20. Servicio de Medicina Interna. Hospital Universitario Virgen del Rocío - Sevilla (Sevilla)

21. Servicio de Cardiología Pediátrica. Hospital Universitario Ramón y Cajal - Madrid (Madrid)

22. Servicio de Neumología/Reumatología. Hospital Universitario Ramón y Cajal - Madrid (Madrid)

23. Servicio de Neumología. Hospital General Universitario de Alicante - Alicante (Alicante)

24. Servicio de Neumología. Hospital Universitario Insular de Gran Canaria - Las Palmas de Gran Canaria (Las Palmas)

25. Servicio de Medicina Interna. Hospital Jerez de la Frontera - Jerez de la Frontera (Cádiz)

26. Servicio de Neumología. Hospital General Universitario de Valencia - Valencia (Valencia)

27. Servicio de Cardiología. Hospital Universitario de Canarias - San Cristóbal de la Laguna (Santa Cruz de Tenerife)

28. Servicio de Cardiología. Hospital Virgen de la Salud - Toledo (Toledo)

29. Servicio de Neumología. Hospital Universitario y Politécnico La Fe - Valencia (Valencia)

30. Servicio de Cardiología. Hospital Universitario Miguel Servet - Zaragoza (Zaragoza)

31. Servicio de Neumología. Hospital Clínico Universitario de Valencia - Valencia (Valencia)

32. Servicio de Cardiología. Hospital Universitario Insular de Gran Canaria - Las Palmas de Gran Canaria (Las Palmas)

33. Servicio de Cardiología. Hospital General Universitario Los Arcos del Mar Menor - San Javier (Murcia)

34. Servicio de Neumología. Hospital Universitario Basurto - Bilbao (Bilbao)

35. Servicio de Cardiología. Hospital del Mar - Barcelona (Barcelona)

36. Servicio de Cardiología. Hospital Universitario Gregorio Marañón - Madrid (Madrid)

37. Servicio de Neumología. Hospital Universitario A Coruña - A Coruña (A Coruña)

38. Servicio de Cardiología. Hospital Clínico Universitario Virgen de la Arrixaca - El Palmar (Murcia)

39. Servicio de Neumología. Hospital Universitario de Gran Canaria Dr. Negrín - Las Palmas de Gran Canaria (Las Palmas)

40. Servicio de Neumología. Hospital Universitario Nuestra Señora de Candelaria - Santa Cruz de Tenerife (Santa Cruz de Tenerife)

41. Servicio de Neumología. Hospital Universitario Vall d´Hebrón - Barcelona (Barcelona)

42. Servicio de Cardiología. Hospital Universitario y Politécnico La Fe - Valencia (Valencia)

43. Servicio de Neumología. Hospital Universitario Son Espases - Palma de Mallorca (Islas Baleares)

44. Servicio de Cardiología. Hospital Universitario Puerta de Hierro Majadahonda - Majadahonda (Madrid)

45. Servicio de Cardiología. Hospital Universitario Infanta Leonor - Madrid (Madrid)
